# Validation of mitotic cell quantification via microscopy and multiple whole-slide scanners

**DOI:** 10.1186/s13000-019-0839-8

**Published:** 2019-06-26

**Authors:** Kazuhiro Tabata, Naohiro Uraoka, Jamal Benhamida, Matthew G. Hanna, Sahussapont Joseph Sirintrapun, Brandon D. Gallas, Qi Gong, Rania G. Aly, Katsura Emoto, Kant M. Matsuda, Meera R. Hameed, David S. Klimstra, Yukako Yagi

**Affiliations:** 1 Department of Pathology, Memorial Sloan Kettering Cancer Center, 1275 York Avenue, New York, NY 10065 USA; 2 Department of Pathology, Nagasaki University Hospital, 1-7-1 Sakamoto, Nagasaki, Nagasaki 8528501 Japan; 3 Center For Devices and Radiological Health, Office of Science and Engineering Laboratories, U.S. Food and Drug Administration, 10903 New Hampshire Avenue, Silver Spring, MD 20993 USA; 4 Department of Pathology, Faculty of Medicine, Alexandria university, 22 El-Guish Road, El-Shatby, Alexandria, 21526 Egypt; 5 Thoracic Service, Department of Surgery, Memorial Sloan Kettering Cancer Center, 1275 York Avenue, New York, 10065 NY USA

**Keywords:** Whole slide imaging, Microscopy, eeDAP, Multiple whole slide scanner, Mitotic cell quantification, Validation study

## Abstract

**Background:**

The establishment of whole-slide imaging (WSI) as a medical diagnostic device allows that pathologists may evaluate mitotic activity with this new technology. Furthermore, the image digitalization provides an opportunity to develop algorithms for automatic quantifications, ideally leading to improved reproducibility as compared to the naked eye examination by pathologists. In order to implement them effectively, accuracy of mitotic figure detection using WSI should be investigated. In this study, we aimed to measure pathologist performance in detecting mitotic figures (MFs) using multiple platforms (multiple scanners) and compare the results with those obtained using a brightfield microscope.

**Methods:**

Four slides of canine oral melanoma were prepared and digitized using 4 WSI scanners. In these slides, 40 regions of interest (ROIs) were demarcated, and five observers identified the MFs using different viewing modes: microscopy and WSI. We evaluated the inter- and intra-observer agreements between modes with Cohen’s Kappa and determined “true” MFs with a consensus panel. We then assessed the accuracy (agreement with truth) using the average of sensitivity and specificity.

**Results:**

In the 40 ROIs, 155 candidate MFs were detected by five pathologists; 74 of them were determined to be true MFs. Inter- and intra-observer agreement was mostly “substantial” or greater (Kappa = 0.594–0.939). Accuracy was between 0.632 and 0.843 across all readers and modes. After averaging over readers for each modality, we found that mitosis detection accuracy for 3 of the 4 WSI scanners was significantly less than that of the microscope (*p* = 0.002, 0.012, and 0.001).

**Conclusions:**

This study is the first to compare WSIs and microscopy in detecting MFs at the level of individual cells. Our results suggest that WSI can be used for mitotic cell detection and offers similar reproducibility to the microscope, with slightly less accuracy.

## Background

For cancer, diagnostic evaluation of histopathology tissue requires the assessment of several parameters, including size, location, the presence of stromal invasion and vascular permeation, and proliferative capacity. These factors are important because they are associated with a variety of critical clinical measures such as malignant potential and therapeutic strategies. Ki-67 quantification, performed using immunohistochemical (IHC) staining, is used as a proliferative marker [1–6]; however, immunohistochemistry (IHC) is expensive and has limited availability in resource-constrained regions. Quantification of proliferative activity by mitotic figures also plays a vital role in predicting tumor proliferation and is often quantitated via hematoxylin and eosin (HE) staining. Guidelines regarding the assessment of tumors include mitotic cell enumeration to determine the malignant potential and prognostic value [5–8]. However, mitotic cell detection also has limitations regarding accuracy and reproducibility [9–12].

Since their first release, digital pathology systems (DPS) have yielded rapid breakthroughs. Several studies have reported that a primary diagnosis based on whole-slide imaging (WSI) was non-inferior to microscopic diagnosis [13–16]. In Europe, several WSI scanners are approved (given the CE mark), and in the US, the US Food and Drug Administration (FDA) has approved the Philips IntelliSite Pathology Solution to be marketed. Additionally, the Pharmaceuticals and Medical Devices Agency in Japan has also approved the Philips system for medical use. DPS has been shown to reduce turn-around times and costs associated with pathological diagnosis [17]. These benefits promote the practical use of DPS for clinical, pathological analysis. However, few studies have reported on the agreement between the use of WSI and microscopy for analysis of histological features at the level of individual cells (e.g., mitotic figure quantification).

Furthermore, DPS enables the use of powerful image processing algorithms for histopathological analysis. Indeed, many automated histomorphologic/cytomorphologic analysis techniques have been commercialized. Additionally, the development of automated mitotic cell detection has also progressed in recent years [18–21]. Thus, it is essential to confirm that mitotic cell detection is accurate and reliable via DPS.

The present study focuses on mitotic cell detection and aims to evaluate mitotic cell detection using WSI with multiple scanners and to determine whether mitotic cell detection using WSI is concordant with microscopy.

## Materials and methods

### Evaluation of the environment for digital and analog pathology (eeDAP)

The US FDA has developed a hardware and software platform called eeDAP. The eeDAP allows for the automated presentation of pre-specified regions of interest (ROIs) or individual cells and cellular features for pathological evaluation [22–24]. The eeDAP can present the ROIs in digital mode using the WSI or in microscope mode using the glass slide on the stage of a microscope. The microscope mode requires a microscope mounted with a camera, motorized stage, and software that registers the stage/slide coordinate system to the WSI coordinate system. The registration accuracy of eeDAP has been shown to be greater than 5 μm.

### Slides, images, and participants

Our study included four HE-stained slides prepared from canine oral melanoma tissues. These slides were part of a pilot study to a larger mitotic figure counting study [25]. The pilot study found these slides to cover a range of mitotic figure counts. We used four slides because the eeDAP system that we used had a maximum capacity of four slides, and we felt that 40 ROIs in total (10 ROIs per slide) would provide some diversity in presentation of mitotic figures from obvious MFs to ambiguous candidates. We also felt that 40 ROIs would not be overly burdensome for the pathologist study participants. The malignant melanoma of canine shows analogous features for human, such as cellular morphology, size, and mitotic figures. Additionally, the slides were readily available to us and did not require any IRB approvals. As a practical matter, there was no observer who felt something wrong with them in comparison with human specimen.

The tumor area on each slide was marked by the slide provider at the National Institutes of Health [25]. Forty ROIs were then randomly selected within the tumor areas marked by a pathologist as being relevant for counting mitotic figures. The ROIs were 200 × 200 μm^2^ field (0.04 mm^2^). For the study, 40 ROIs from four slides were randomly selected within the tumor area of the tissue and reviewed by a pathologist. The eeDAP system allows for the automated presentation of the same ROI to observers using a microscope or a WSI digitized by four types of WSI scanners at two institutes:

- Aperio AT2 (Leica Biosystems Inc., Buffalo Grove, IL, USA), 40× (0.25 μm/pixel), NA 0.75; we used two machines of this type, belonging to the National Institute of Health, and the Memorial Sloan Kettering Cancer Center (MSKCC), respectively.
- NanoZoomer 2.0-HT (Hamamatsu Photonics K.K., Hamamatsu, Shizuoka, Japan), 40× (0.23 μm/pixel), NA0.75, belonged to the MSKCC.
- Pannoramic 250 Flash III (3DHISTECH Ltd., Budapest, Hungary), 40× (0.13 μm/pixel), NA0.95, belonged to the MSKCC.

Five observers were asked to identify all mitotic cells in the ROIs with microscopy and with WSI. The eeDAP system presented the whole slide images with the ROI outlined by a square using Leica Aperio ImageScope (v12) whole slide viewer software. This software allows for changing magnification and panning, but it was not necessary. On the microscope, eeDAP drives the stage to the specific ROI, which is outlined by a reticle in the eyepiece. The pathologist is encouraged to focus the microscope and discouraged from moving the stage laterally. We call these “candidate” mitotic cells. All participants were pathologists with an experience of 15, 14, 8, 6, and 5 years. Four of them had participated in other validation studies of primary diagnosis using WSI, and they had been trained based on guidelines for the validation study of WSI submitted by College of American Pathologists at that time. Another pathologist had been engaged in research for automated histological analysis using WSI. Therefore, all pathologists participated in this study had excellent proficiency in histological observation via WSI. Each observer investigated mitotic figures using 5 modalities (4 types of WSI scanner and brightfield microscope). Each modality was evaluated in a separate session. As such, each ROI only appeared once in each session. Wash out time was longer than 2 weeks between sessions.

### Definition of mitosis

Criteria for mitosis included the loss of the nuclear membrane, accompanied with chromatin condensation, forming the mitotic apparatus. Stages of mitoses that were included were representative of prophase through anaphase. Two consecutive daughter cells with newly formed nuclear membranes signified the end of mitosis (i.e., telophase) and were not considered mitotic cells in this study.

### Definition of “ground truth” of mitosis

Ground truth was defined on the basis of microscopic observations. First, the consensus team included all candidates that were detected by more than four observers via microscopy as ground truth. The consensus team comprised certified experienced pathologists (two with 13 years and one with 9 years of experience). The truthing panel also considered all other candidates, regardless of modality and initial agreement results. The truthing panel did this in a group setting using a digital microscope (VisionTek; Sakura Finetek Japan Co., Ltd., Tokyo, Japan) to determine which candidates were true mitotic figures (MFs).

### Statistical analysis

We assessed three types of agreements: 1) inter-observer agreement within each viewing mode, 2) intra-observer agreement between the different viewing modes, and 3) accuracy, defined as an agreement between detections in each viewing mode and ground truth. Inter- and intra-agreement was analyzed using Cohen’s Kappa statistics, giving the 2 × 2 tables of the mitosis positive and negative determinations of all candidate mitotic cells. Following Landis and Koch [26], we categorized the Kappa values as slight (≧ 0, < 0.2), fair (≧ 0.2, < 0.4), moderate (≧ 0.4, < 0.6), substantial (≧ 0.6, < 0.8), and almost perfect agreement (≧ 0.8).

Intra-observer agreement between the scanner and microscope data was also analyzed with Bland-Altman plots and related summary statistics. For each modality we plot the differences in log counts between the paired scanner and microscope data for each pathologist against the average of each pair [27]. The log transform stabilizes the variance in the count differences as a function of the mean [20]. The summary statistics include the mean differences in log counts and the standard deviation of the log-count differences (uncertainty). Twice the standard deviation of the log-count differences above and below the mean give the limits of agreement (LA). LA are similar to but different from confidence intervals, which typically quantify uncertainty in a mean. For this analysis, we counted all the cells marked as MFs for each reader in a WSI. This aligns with what is done in clinical practice. Therefore, we have four counts for each reader and modality. The uncertainties estimated in this Bland-Altman analysis account for the variability from the pathologists and the correlations that arise when the pathologists evaluate the same cases, a so-called multi-reader multi-case analysis [28].

Accuracy was analyzed using the average of sensitivity and specificity, giving the 2 × 2 tables of true and false MFs vs. positive and negative determinations of all candidate MFs. Sensitivity is defined as the number of MFs detected by an observer divided by the number of true MFs. Specificity is defined as one minus the false-positive fraction, where the false-positive fraction is the number of false MFs that were positively marked, divided by the total number of false MFs. This average is equivalent to the area under the receiver operating characteristic curve for binary scores and is proportional to Youden’s index [29, 30]; it is also correlated with Cohen’s Kappa [31]. We reported the accuracy for each reader and modality and then the average over readers for each modality. We also performed a multiple-reader multiple-case (MRMC) analysis of reader-averaged accuracy using the Obuchowski-Rockette (OR) method [32, 33]. This method takes as input the covariances between the AUCs from all the reader by modality combinations (five readers times five modalities). These covariances account for within-slide correlation between measurements obtained on ROIs within the same slide [34, 35].

In this study, to determine the statistical significance of four accuracy comparisons (the microscope compared to each of the four scanners), we performed the sequentially rejective Bonferroni test, with alpha = 0.05 [36] . All the MRMC analyses were performed with the iMRMC application (version 4.0) developed by the US FDA [37].

## Results

All 5 observers detected a total of 155 candidate mitotic cells, using both WSIs and microscopy. The counts by all observers for each observation method are shown in Table 1. Using microscopy, 29 potential candidate mitotic cells were detected by all five observers, 8 candidates by four observers, 17 candidates by three observers, 13 candidates by two observers, and 28 candidates by one observer. The remaining 60 candidates were not detected by microscopy but were detected by WSI. Of these 60 candidate mitotic figures, the truthing panel determined that four of them were true mitotic figures, and there was otherwise little-to-no consensus. Within a scanner, only one candidate was marked by three pathologists and only six candidates were marked by two pathologists. The remaining candidate mitotic figures were marked by only one pathologist (within a scanner).

**Table 1:** Enumeration of candidate mitotic figures (number and percentage in total 155 candidate mitotic figures)

	Scanner A	Scanner B	Scanner C	Scanner D	Microscope
Observer 1	43 (28%)	57 (37%)	35 (23%)	42 (27%)	41 (26%)
Observer 2	64 (41%)	49 (32%)	28 (18%)	41 (26%)	66 (43%)
Observer 3	55 (35%)	39 (25%)	36 (23%)	60 (39%)	51 (33%)
Observer 4	34 (22%)	43 (28%)	39 (25%)	34 (22%)	64 (41%)
Observer 5	35 (23%)	48 (31%)	39 (25%)	46 (30%)	60 (39%)
Ground truth					74 (47.1%)

**Fig. 1:**
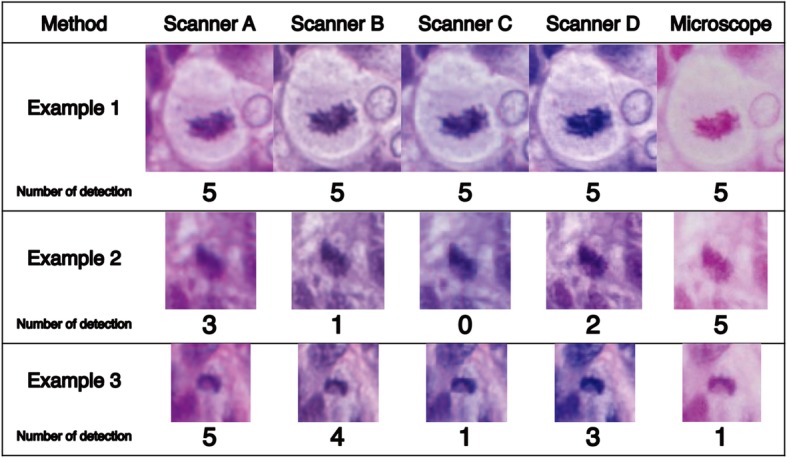
Examples of ground truth in mitotic cell imaging. Three examples of ground truth for mitotic cell imaging are shown. Example 1: images of mitotic cells obtained by all observers using all observation methods included microscopy; example 2: no participant detected mitotic cells, using scanner C; example 3: only one observer detected mitotic cells upon microscopic examination

For ground truth, 37 candidates were detected by more than four observers; these were considered true mitotic cells. The consensus team evaluated the other candidates, and 74 were finally confirmed as true mitotic cells. In Fig. 1, three ground truth mitotic figures are shown: 1) example 1, confirmed as mitotic cells by all observers using all observation methods, 2) example 2, confirmed by none of the observers using scanner C, and 3) example 3, confirmed by only one observer via microscopy. All Kappa coefficients of inter-observer agreement were “substantial” to “almost perfect” (Table 2). Furthermore, all intra-observer agreements were “substantial” to “almost perfect” (Table 3).

**Table 2:** Inter-observer agreement in each observation method (Kappa coefficient)

	Scanner A	Scanner B	Scanner C	Scanner D	Microscope
Observer 1 vs 2	0.677	0.770	0.814	0.815	0.735
Observer 1 vs 3	0.775	0.790	0.872	0.745	0.833
Observer 1 vs 4	0.885	0.788	0.852	0.865	0.779
Observer 1 vs 5	0.864	0.799	0.879	0.820	0.781
Observer 2 vs 3	0.621	0.807	0.834	0.667	0.742
Observer 2 vs 4	0.693	0.763	0.840	0.791	0.723
Observer 2 vs 5	0.699	0.802	0.840	0.786	0.666
Observer 3 vs 4	0.773	0.850	0.818	0.819	0.831
Observer 3 vs 5	0.765	0.862	0.845	0.785	0.789
Observer 4 vs 5	0.886	0.833	0.864	0.905	0.743

**Table 3:** Intra-observer agreement in each observation method (Kappa coefficient)

	Observer 1	Observer 2	Observer 3	Observer 4	Observer 5
Scanner A vs Microscope	0.835	0.677	0.784	0.748	0.830
Scanner B vs Microscope	0.775	0.729	0.861	0.763	0.797
Scanner C vs Microscope	0.824	0.717	0.814	0.738	0.726
Scanner D vs Microscope	0.815	0.664	0.804	0.776	0.799
Scanner A vs B	0.816	0.745	0.777	0.858	0.808
Scanner A vs C	0.851	0.704	0.744	0.859	0.892
Scanner A vs D	0.856	0.594	0.728	0.880	0.742
Scanner B vs C	0.792	0.784	0.939	0.891	0.848
Scanner B vs D	0.810	0.709	0.852	0.871	0.761
Scanner C vs D	0.831	0.772	0.832	0.872	0.754

**Fig. 2:**
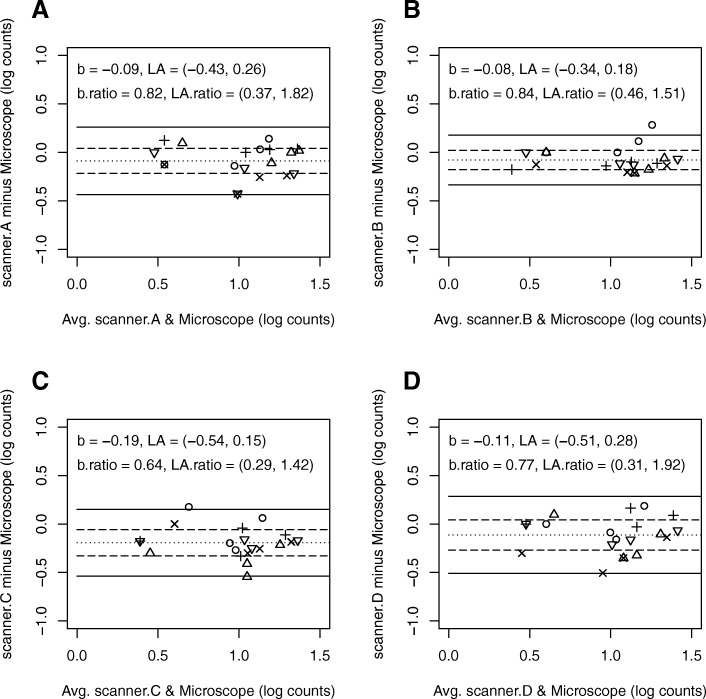
Bland-Altman plots of within-reader differences in log (base 10) counts between each scanner (**a**, **b**, **c**, **d**) and the microscope. Each symbol corresponds to a different reader. The dotted line in each plot is b, the mean difference in the log counts. The dashed lines show the 95% MRMC confidence interval for b. The solid lines show the MRMC limits of agreement (LA). We map b and LA to ratios of counts with the inverse log (base 10) transformation, or 10ˆb, 10ˆLA

In Fig. 2, we show the within-reader Bland Altman plots comparing log-count differences from the scanners to those with the microscope. The biases observed in the log counts show that the pathologists marked fewer MFs with the scanners compared to the microscope. They marked between 16 to 36% fewer on average and 70% fewer in some cases.

**Fig. 3:**
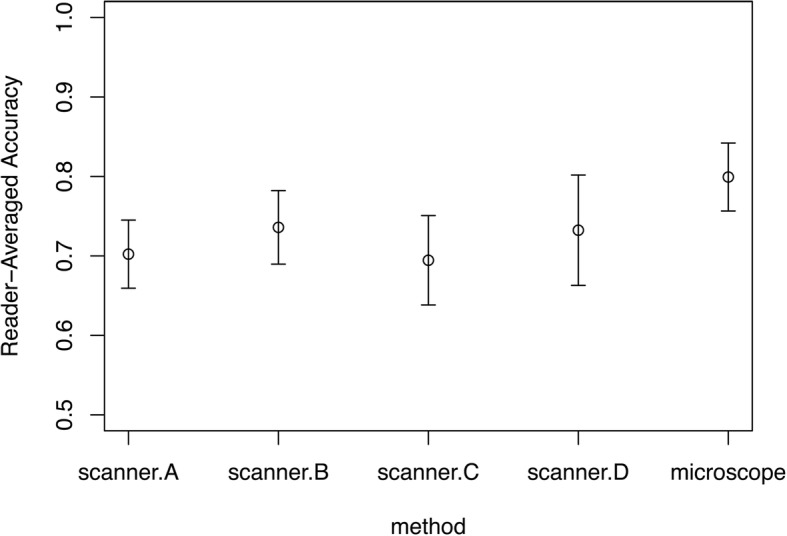
Accuracy (average of sensitivity and specificity) for each viewing mode averaged over all the readers with 95% confidence intervals. The asterisks indicate that the difference in accuracy of the viewing mode compared to that of microscopy is statistically significant. All analyses account for the correlations and variability from the readers reading the same ROIs

To compare all detected mitotic cell candidates with ground truth, we analyzed accuracy which is the average of sensitivity and specificity. Accuracy was between 0.631 and 0.842 across all readers and modes (Table 4, Fig. 3). After averaging over readers for each detection method, we found [36] that mitosis detection accuracy of each of the three scanners, A, B, and C, was significantly less than that of the microscope.

**Table 4:** Accuracy for all readers and observation methods

	Scanner A	Scanner B	Scanner C	Scanner D	Microscope
Observer.1	0.713	0.743	0.685	0.706	0.764
Observer.2	0.700	0.715	0.631	0.648	0.778
Observer.3	0.704	0.738	0.717	0.802	0.806
Observer.4	0.691	0.726	0.699	0.717	0.842
Observer.5	0.698	0.754	0.738	0.785	0.802
Average	0.701	0.735	0.694	0.732	0.798
SE	0.021	0.023	0.028	0.035	0.021
95% CI	(0.659, 0.743)	(0.689, 0.780)	(0.636, 0.752)	(0.653, 0.810)	(0.754, 0.842)
*p*-value	0.001*	0.009*	0.001*	0.062	

Accuracy refers to the average of sensitivity and specificity. SE, standard error; CI, confidence interval. The *p*-value corresponds to a two-sided hypothesis test comparing reader-averaged accuracy with each scanner viewing mode to the accuracy of the microscope. The *p*-values of the four hypotheses are compared following the sequentially rejective Bonferroni test with alpha = 0.05 [33]. Statistical significance is indicated with an asterisk *. All analyses account for the correlations and variability from the readers reading the same ROIs, and the correlations arising from MFs contained within the same slides

## Discussion

This study is the first, to our knowledge, to use WSIs of multiple vendors and glass microscopy to evaluate mitotic cells identification. Most previous studies used only one type of scanner to compare WSI and microscopy. In this study, we hypothesized that it is possible to assess “a certain WSI scanner of a certain manufacturer” with a task-based feature study along with the conventional examination. Hence, we used four types of scanners (all having almost the same scanning capabilities) to validate WSI as “instruments” for pathological analysis. Ultimately, inter-observer agreements within all presentation modes (microscopy and all WSI sets) were “substantial” or greater, and intra-observer agreements between all observation methods displayed a similar trend. One interesting finding worth investigating with a larger study is that the pathologists found fewer mitotic figures on the scanners than on the microscope. The present results suggest that WSI is a viable “instrument” to detect mitotic cells because it was reproducible in comparison with microscopy.

Furthermore, we attempted to evaluate mitotic cell detection in detail. Although evaluation using entire slide glass is a viable method for enumerating mitotic cells, it is not suitable for microscopic imaging of individual mitotic cells because of difficulties of annotating ROIs or target cells upon microscopic observation. The eeDAP system enabled us to evaluate mitosis at the level of individual cells. Currently, only one previous study has compared WSI and microscopy [38]. Owing to this new strategy, we could determine the ground truth of mitosis starting with the candidate mitotic cells detected by all observers. We found that it is not feasible to detect ground truth with a single pathologist, using either WSI or microscopy alone, because no observer and observation method could identify all the 74 ground truth mitotic cells. These results suggest that microscopy is also an imperfect method to detect mitotic cells.

Interestingly, microscopy was measurably more accurate than 3 of the 4 WSIs. Despite its simplicity, microscopy for detecting mitotic cells provides important information regarding malignant potential and therapeutic strategies for various tumors [5–8]. This implies that discrepancies in mitotic cell detection may affect pathologic interpretations and thus potentially patient care. One explanation for why the accuracy of the microscope was better is that the microscope allows the pathologist to focus on different z-planes. In reality, one of the WSI sets used herein was out of focus and warranted re-scanning. Observers were concerned that the re-scanned WSIs were slightly opaque, although they could determine the histological type. Although 20× scanned WSIs are reportedly viable for histological diagnosis [16], it is expected that the resolution of WSIs scanned at 20× are insufficient to observe mitotic cells. Because pathologists often evaluate images at 40× magnification to detect mitotic cells, the image is likely to be somewhat opaque when the WSIs are magnified digitally from 20× to 40×. In addition to the capacity of the scanner, inadequate maintenance of scanners probably leads to problems in focusing. Regarding scanner C, the light source contained a covering of dust, and the glass slide stage was determined to be unstable upon periodic inspection after this study. Insufficient maintenance does not allow for optimal performance of the scanner; hence, maintenance of the scanner is also essential for WSI-based diagnosis.

Z-stacking or multilayer scanning is a viable method to resolve the issue regarding z-plane focusing. Current WSI scanners can perform z-stacking or multilayer scanning, which has enabled pathologists to adjust the focus, similar to a microscope. However, considering the number of layers and the distance between each layer, it might be that z-stacking/multilayer scanning is not sufficient for observation, owing to disadvantages such as large file sizes and extended scanning time. Adjustment of the numerical aperture (NA) may prove a promising method to resolve this issue. It is needless to say that pixel resolution also has an impact on image quality. NA is also associated with the optical resolution of the microscope and WSI; a higher NA leads to better image quality. In fact, scanner D had a higher pixel resolution (0.13 μm/pixel) and higher NA (0.95) than other scanners. Hence, it is possible to resolve this issue by employing a WSI scanner with higher pixel resolution and NA.

The condition of glass slides is also an important consideration. Generally, staining intensity of the slides becomes pale upon exposure to light. In reality, the staining intensity was reduced via microscopy when we digitized WSI images using scanner D and re-digitized using scanner C; these slides were scanned after repeated digitization by other scanners and microscopic observation. Although we considered repeating HE staining after bleaching, it was not performed because it is difficult to reproduce the original color tone completely, although re-staining will subsequently be performed. The color-tone corrective function of WSI viewers might resolve this issue; however, this was not performed in this study.

These issues about mitotic figure detection using WSI also affect computational pathology. Automated evaluation of IHC and liquid-based cytological specimens are used in clinical practice, and several studies have reported the use of automated mitotic cell detection [18–21]. Precise recognition of mitotic cells is necessary to develop accurate automated systems to detect mitotic cells. First, the reproducibility of mitotic cell enumeration by pathologists is controversial [9–12], and the present results also reveal that microscopic mitotic cell detection by pathologists would not have encompassed entirely ground truth as defined. Successful development of an automated mitotic cell detection system should contribute to diagnosis and therapy. To convert this to reality, we should understand and recognize the specific limitation regarding mitotic cell detection using WSI, as shown in the present study.

In this study, the arbitrary decision regarding the ground truth for comparisons represented a limitation, which was a critical and problematic issue. IHC for phosphohistone H3 (pHH3) is a popular method to detect mitotic cells; furthermore, pHH3 is an essential marker to differentiate mitosis from apoptosis [39]. pHH3 reportedly correlated with patient prognosis [40] and improved inter-observer reproducibility of mitotic cell detection [41]. IHC for pHH3 appears to be an excellent tool for mitotic cell detection; however, it is not easy to evaluate IHC results owing to background noise, false positive staining, and different staining intensities due to sample condition. These issues make it difficult for pathologists to do an accurate assessment. Furthermore, IHC for pHH3 was suggested not to be a substitute for detecting mitotic cells via HE staining [42]. Hence, performing both IHC and HE staining in the same slide or serial slides of the same tissue can effectively detect mitotic cells. However, this was not performed because all slides had already been prepared before the study. If IHC is performed for HE-stained slides after bleaching, it is not possible to assess different staining methods for the same slide with the microscope. In the current study, we identified candidate mitotic figures by many pathologists using the microscope and four WSIs, and then a consensus team reviewed the candidates.

The data and analysis scripts are available online (https://github.com/DIDSR/iMRMC/wiki/Tabata2019_comparingScannersMFcounting).

## Conclusion

To the best of our knowledge, this study is the first to use multiple scanners and microscopy to evaluate the detection of mitotic cells at the level of individual cells. Our results suggest that certain WSI scanners are viable “instruments” to detect mitotic cells with similar reproducibility as the microscope but with potential loss of accuracy. As such, care should be taken when using WSI to detect mitotic cells in pathological diagnosis and developing an algorithm for mitotic detection as the accuracy level is slightly inferior to microscopy. Appropriate maintenance and management of both WSI scanners and histological slides can help optimize the performance of DPS. Further development and application of WSI are expected to yield further advancements in the future.

## Data Availability

The datasets used and analyzed during the current study are available from the corresponding author on reasonable request.

## Abbreviations

DPS: Digital pathology systems
eeDAP: evaluation of the environment for digital and analog pathology
HE: hematoxylin and eosin
IHC: Immunohistochemistry
MFs: Mitotic figures
MRMC: Multiple-reader multiple-case
MSKCC: Memorial Sloan Kettering Cancer Center
NA: Numerical aperture
pHH3: phosphohistone H3
ROI: Regions of interest
US FDA: the US Food and Drug Administration
WSI: Whole-slide imaging
